# Letter to the editor regarding “Risk factors for incomplete excision of cutaneous squamous cell carcinoma: A single-center study performed on 1082 excisions in Northern Italy”

**DOI:** 10.1016/j.jpra.2025.08.010

**Published:** 2025-08-07

**Authors:** Shashank Dokania, Parth Aphale, Himanshu Shekhar

**Affiliations:** Research and Development Cell, Dr. D.Y. Patil Vidyapeeth (Deemed to be University), Pimpri, Pune, Maharashtra, India

To the Editor,

We read with great interest the article by van Helmond et al.^1^ titled *“Risk factors for incomplete excision of cutaneous squamous cell carcinoma: a single-center study performed on 1082 excisions in Northern Italy”* published in *JPRAS Open* (2025;44:316–330). The authors have undertaken an important and commendable effort to investigate tumor-, procedural-, and patient-related factors that may contribute to incomplete excision rates in cutaneous squamous cell carcinoma (cSCC), leveraging one of the largest surgical cohorts reported from a plastic surgery perspective. The study contributes meaningfully to existing literature by highlighting the role of certain tumor characteristics such as location (ear, peri‑orbital), size, thickness, ulceration, differentiation grade, and infiltration level as significant predictors of incomplete excision. Moreover, the statistically significant association between the absence of a prior diagnostic biopsy and incomplete margins underscores the procedural vigilance necessary during preoperative planning. These findings are in alignment with systematic reviews that emphasize lesion complexity and anatomical site as critical determinants of surgical adequacy.^2^

Nonetheless, there are methodological considerations that deserve further discussion. One notable concern is the exclusion of multivariable logistic regression despite identifying numerous significant univariable predictors. The authors acknowledge this limitation, citing the large number of significant variables. However, omitting multivariable analysis restricts our understanding of the independent predictive strength of each factor when adjusted for potential confounders. As emphasized by Marsidi et al.^3^ comprehensive multivariate modeling remains a necessary step in deriving risk models applicable to clinical practice.

Additionally, the study defines incomplete excision solely based on the presence of tumor cells at the margin of the main specimen, irrespective of additional samples taken intraoperatively. While this adheres to standard pathology protocols, the association between “non-tumor-free” status in additional samples and incomplete excision highlights a disconnect between intraoperative suspicion and histopathological outcomes. A more nuanced analysis, possibly stratifying these events, could refine operative decision-making and underscore the clinical utility of frozen section analysis or Mohs micrographic surgery (MMS), which is notably absent in the study center's protocol.^4^

In summary, the study by van Helmond et al.^1^ provides valuable insights into surgical quality and risk stratification in the treatment of cSCC. Future research that incorporates multicenter data, multivariate modeling, and comparative technique-based analysis (WLE vs. MMS) would offer greater generalizability and guide individualized treatment approaches in dermato-oncology.

## CRediT authorship contribution statement

SD: Analysis, Interpretation, Writing the manuscript. PA: Data acquisition, Writing the manuscript. HS: Conception and Design, Final review, Conceptualization.

## Declaration of competing interest

The authors declare that they have no known competing financial interests or personal relationships that could have appeared to influence the work reported in this paper.
